# Supplementary material for: Optimal features for auditory categorization
Source: Nat Commun. 2019 Mar 21;10:1302. doi: 10.1038/s41467-019-09115-y (PMC6428858; doi:10.1038/s41467-019-09115-y)
Supplement: Supplementary file 1 — Supplementary Information [file 41467_2019_9115_MOESM1_ESM.pdf]

Supplementary Material

Optimal Features for Auditory Categorization

Shi Tong Liu, Pilar Montes-Lourido, Xiaoqin Wang, Srivatsun Sadagopan

Supplementary Note

MIF-based reconstruction of call stimuli

The observation that an MIF-based approach successfully generalizes across production variability implies that most calls belonging to a category will contain one or more of the MIFs. Therefore, we asked how well calls could be reconstructed based on MIFs alone, using twitters as a specific example. To do so, we detected model twitter MIF neuron spiking as described in the main text to the 500 training and 500 test twitters, and convolved these spike times with an alpha function (with a time constant of 20 ms) to detect the peak locations of twitter MIFs within a twitter (Supplementary Fig. 5A). We then placed copies of MIF cochleagrams at these peak locations, or added copies of MIF cochleagrams to previously placed feature cochleagrams. The final summed cochleagram was taken to be the reconstructed call (Supplementary Fig. 5B). We evaluated the accuracy of reconstruction as the NCC value at zero lag. The mean reconstruction accuracy was 0.69 (Supplementary Fig. 5C), suggesting that MIFs were indeed common denominators across twitter calls produced by different animals.

Supplementary Discussion

Factors contributing to the success of the MIF-based approach

Three factors were critical in the design and implementation of our approach. First, focusing on a behaviorally critical task (call categorization), and choosing model species with rich vocal repertoires and behaviors (marmosets and guinea pigs) allowed us to clearly identify a computational goal of cortical processing – call categorization. Previous

experiments using both electrophysiological and imaging techniques[1 – 8], showing an increase in cortical resources allocated to call processing, validate our choice of call categorization as a critical computational goal in vocal animals. Second, our analyses were based on a large sample of calls recorded from a large number of animals. From this data set, we deliberately oversampled a large number of initial potential features. This ensured that the full extent of production variability was represented in this data set. Third, the greedy search algorithm efficiently identified informative features from a training data set of a few hundred calls. Since clean and labelled training data sets are laborious to generate, the efficiency of greedy search provided a significant methodological advantage.

Limitations of greedy search and MIF-based classification

In this study, we used greedy search and pairwise maximization of information to find optimal features. However, it is possible that the greedy search algorithm does not find an optimal solution because of its inability to overcome local maxima. We do not think this is the case because: 1) the model performs at high accuracy levels, leaving little room for significant improvements, 2) we could arrive at similar sets of MIFs and achieve similar performance levels from different initial feature sets, specifically when highly informative features were excluded (Supp. Fig. 3), and 3) we could match or outperform other machine learning based algorithms for marmoset call classification[9]. Therefore, the implemented greedy search algorithm likely converges at a true optimal solution.

Our MIF-based approach has two limitations. First, the number of auditory tasks that an animal is potentially required to solve is ill-defined. While we mitigate this limitation by choosing ethologically critical tasks such as call categorization, it is likely that we are

only probing a small subset of all behaviorally relevant auditory tasks. Consequently, while a subset of neurons in auditory cortex match predictions from our model for call and caller classification, developing a larger bank of natural auditory behavior (for example, predator sounds versus neutral sounds) will allow us to model and predict a larger fraction of cortical responses. Second, our model derives features from the auditory nerve representation of stimuli. It is well-known that this representation is transformed more than once before impinging on cortical neurons. Therefore, the actual representation from which cortical neurons detect features are not accurately modeled here. This limitation arises from the current lack of predictive models for central auditory processing stages. It is possible that the performance of our algorithm will increase if we could accurately model other sub-cortical processing stages.

Alternative models

Recently, theoretical efforts have been directed at learning invariant representations from small training sets using unsupervised methods[10]. In this model, image 'signatures' which serve as a proxy for the probability distribution of an image and its transformations are learnt by leveraging the time correlations of image transformations in the real world to label image identity. Image signatures can be computed by complex cell-like units using Hebbian learning rules. This model predicts that a similar computation might occur in auditory cortex. The MIFs that we have derived for call categorization are similar to the image signatures in that they serve as a proxy for the probability distribution of a sound category that has been subjected to production variability. Indeed, vocalizations can be viewed as multivariate probability distributions along multiple call parameters, and MIFs could serve as the 'gist' of a call category around which these

variations occur. Similar to image signatures, MIFs seem to be computed by superficial-layer auditory cortex neurons. However, differences arise in how MIFs are learnt. Although small sample sizes are adequate, unlike image signatures that are learnt by observing image transformations over time, explicit labeling of the class of input examples is necessary for learning the MIFs of calls. Conceptually, whereas image signatures are learnt by observing within-category transformations, MIFs are learnt by contrasting the distributions of sound categories.

Alternative experimental approaches

Previous experimental studies have described call selectivity primarily using two methods: 1) characterization of neural tuning along an exhaustive list of call parameters[11], and 2) characterizing call tuning as tuning for regions of the modulation spectrum[12 – 14]. In the former study, marmoset calls were parametrized along multiple acoustic dimensions. Some of these parameters were common to all call types, such as the length or dominant frequency of a call. The more distinguishing parameters, however, were unique to individual call types, such as the inter-phrase interval for twitters, or sinusoidal frequency modulation rate for trills. Neural tuning to calls was described using tuning to these parameters but did not use the same set of parameters across call types. In our study, different MIFs are used for classification of different call types, but MIFs are parametrized along the same axes – bandwidth and integration window, allowing for a uniform basis for comparisons. In the latter set of studies, neural tuning for birdsong was described using selectivity for specific frequency and temporal modulations. In this case, tuning could be expressed in a unified stimulus space (of spectral- and temporal modulation rates). Both these methods, however, serve to describe neural tuning, and

not to explain why tuning to certain parameters or regions of modulation space are necessary in the first place. Our results suggest that generating selectivity for task-relevant features explains why selectivity for stimulus parameters arises in the first place. In a recent study, a combination of the above approaches was used in conjunction with statistical classifier techniques to achieve caller identification for macaque coo calls[15]. Caller identification could not be achieved using a single feature alone, where feature referred to a parameter such as fundamental frequency, duration, or location in the modulation spectrum. Rather, a combination of cues was required for high caller identification performance. Our study differs from this study in that our definition of 'feature' is non-parametric, our goal is to generalize over individual identity, and features are contrastive and task-dependent. But similar to this study, a single feature alone was insufficient for call categorization in our study as well.

Supplementary References

1. Rauschecker JP, Tian B. Mechanisms and streams for processing of "what" and "where" in auditory cortex. Proc Natl Acad Sci USA 97:11800-11806 (2000).
2. Tian B, Reser, D, Durham A, Kustov A, Rauschecker JP. Functional Specialization in Rhesus Monkey Auditory Cortex. Science 292:290-293 (2001).
3. Romanski LM, Averbeck BB. The primate cortical auditory system and neural representation of conspecific vocalizations. Annu Rev Neurosci. 32:315-346 (2009).
4. Grimsley JM, Shanbhag SJ, Palmer AR, Wallace MN. Processing of communication calls in guinea pig auditory cortex. PLoS One 7:e51646 (2012).

5. Fukushima M, Saunders RC, Leopold DA, Mishkin M, Averbeck BB. Differential coding of conspecific vocalizations in the ventral auditory cortical stream. J Neurosci 26:4665-4676 (2014).
6. Petkov CI, Kayser C, Steudel T, Whittingstall K, Augath M, Logothetis NK. A voice region in the monkey brain. Nat Neurosci 11:367-374 (2008).
7. Perrodin C, Kayser C, Logothetis NK, Petkov CI. Voice cells in the primate temporal lobe. Curr Biol 21:1408-1415 (2011).
8. Sadagopan S, Temiz-Karayol NZ, Voss HU. High-field functional magnetic resonance imaging of vocalization processing in marmosets. Sci Rep 5:10950 (2015).
9. Turesson HK, Ribeiro S, Pereira DR, Papa JP, de Albuquerque VHC. Machine learning algorithms for automatic classification of marmoset vocalizations. PLoS One 11: e0163041 (2016).
10. Anselmi F, Leibo JZ, Rosasco L, Mutch J, Tacchetti A, Poggio T. Unsupervised learning of invariant representations. Theor Comput Sci 633: 112 – 121 (2016).
11. DiMattina C, Wang X. Virtual vocalization stimuli for investigating neural representations of species-specific vocalizations. J Neurophysiol 95:1244-1262 (2006).
12. Hsu A, Woolley SM, Fremouw TE, Theunissen FE. Modulation power and phase spectrum of natural sounds enhance neural encoding performed by single auditory neurons. J Neurosci. 24:9201-9211 (2004).

13. Woolley SM, Fremouw TE, Hsu A, Theunissen FE. Tuning for spectro-temporal modulations as a mechanism for auditory discrimination of natural sounds. Nat Neurosci 8:1371-1379 (2005).

14. Stowell D, Plumbley MD. Large-scale analysis of frequency modulation in birdsong data bases. Methods in Ecology and Evolution 5:901-912 (2014).

15. Fukushima M, Doyle AM, Mullarkey MP, Mishkin M, Averbeck BB. Distributed acoustic cues for caller identity in macaque vocalization. R Soc open sci 2: 150432 (2015).

Supplementary Figures

Liu ST, Montes Lourido MP, Wang X, and Sadagopan S.

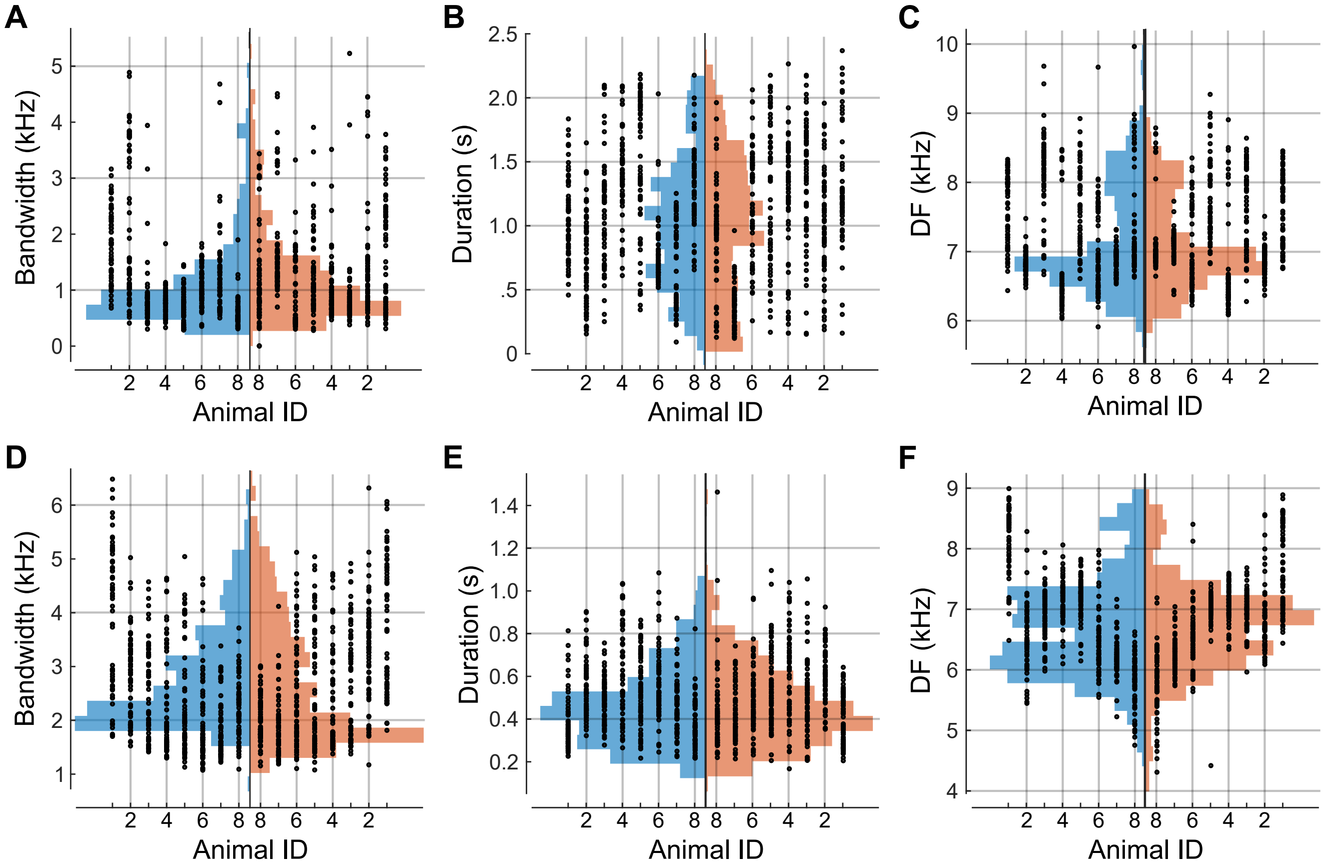

Supplementary Figure 1: Production variability of major marmoset call types. (A-C) Production variability of phee calls quantified along various parameters: **(A)** bandwidth, **(B)** duration, and **(C)** dominant frequency. Dots depict parameter values for single calls, and histograms indicate the overall distribution of these parameters, split into the training (blue) and testing (red) sets. **(D-F)** Production variability of trill calls quantified as in (A-C).

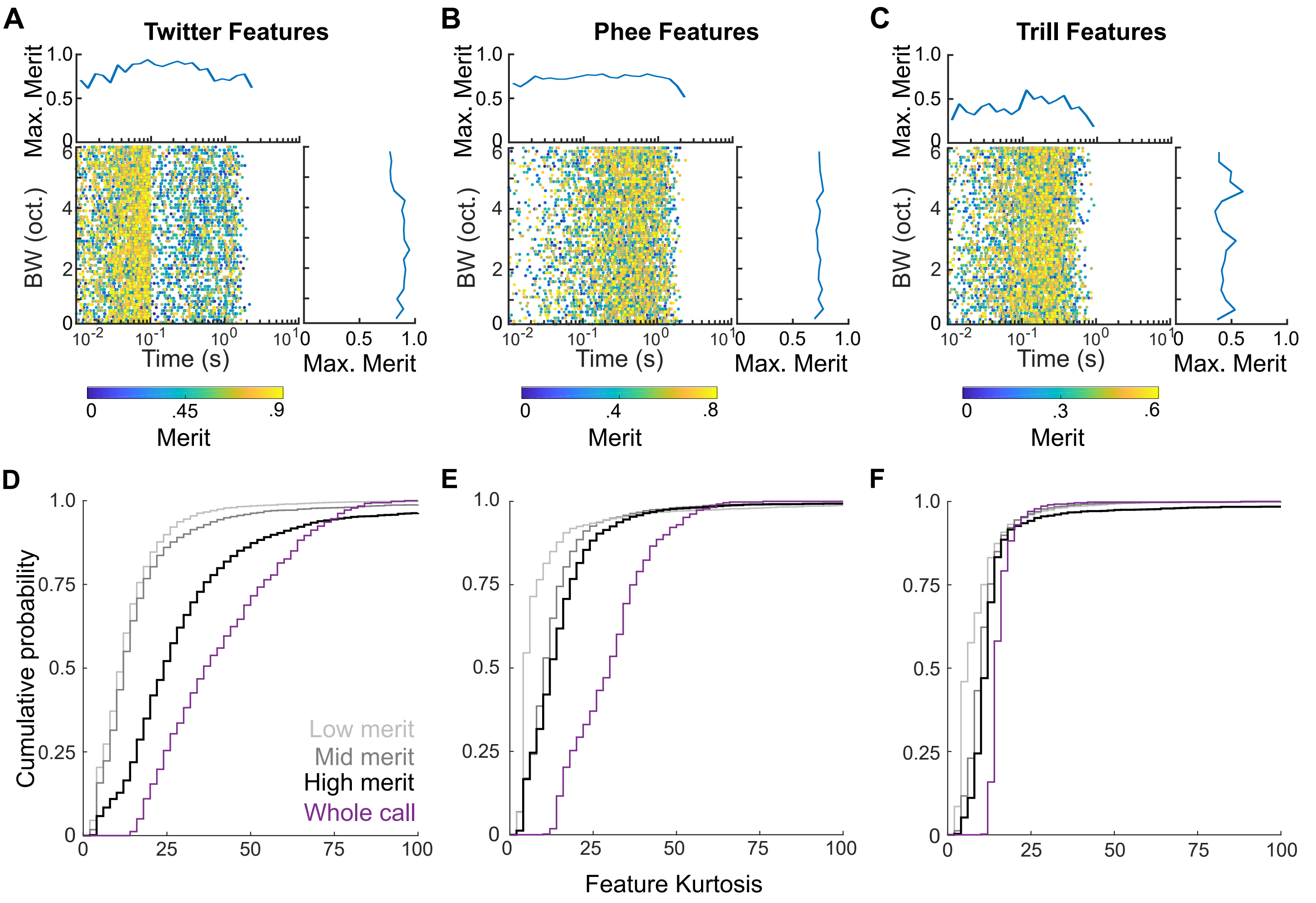

Supplementary Figure 2: Information content, complexity, and size of all initial random features. Scatter plot of all 6000 features generated for each call type: twitter **(A)**, phee **(B)**, and trill **(C)**, as a function of their bandwidth and temporal extent. Color scale corresponds to the merit of each feature. Marginal histograms depict the maximum merit in each time- or bandwidth-bin. **(D-F)** Features of high merit for classifcation tend to be of intermediate complexity. Merit vs complexity plot of all randomly generated twitter **(D)**, phee **(E)**, and trill **(F)** features. Feature complexity is estimated to be proportional to the reduced kurtosis of the distribution of activity within a feature or call. In these plots, low- or mid-merit features (defined as the bottom 33-%ile (light gray) and 33rd - 66th %-ile (dark gray)) show distributions of low kurtosis values. Whole calls show high kurtosis values (purple). Across call types, high-merit features (top 33%-ile) show intermediate kurtosis values, indicating that high-merit features are of intermediate complexity.

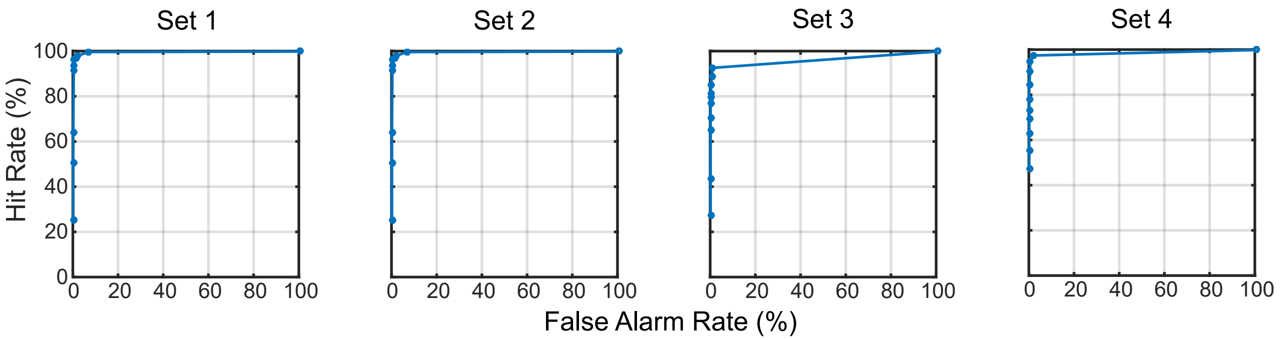

Supplementary Figure 3: Similar classification performance obtained using distinct MIF sets. ROC curves for twitter classification using four successive iterations of MIFs, generated by removing all MIFs from the previous set, and selecting MIFs from the remaining features. High performance demonstrates that feature space was adequately sampled, and that the algorithm was not stuck in local maxima.

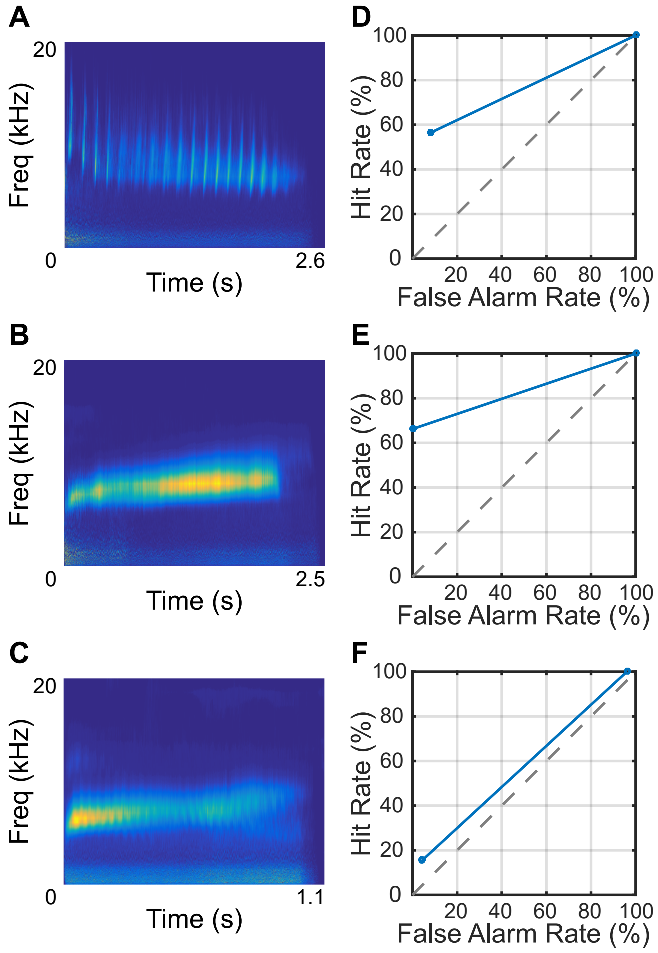

Supplementary Figure 4: Classification using average calls. An average twitter **(A)**, trill **(B)**, and phee **(C)** constructed by aligning and averaging over the calls. **(D-F)** Classification performance using the average call as the single informative feature.

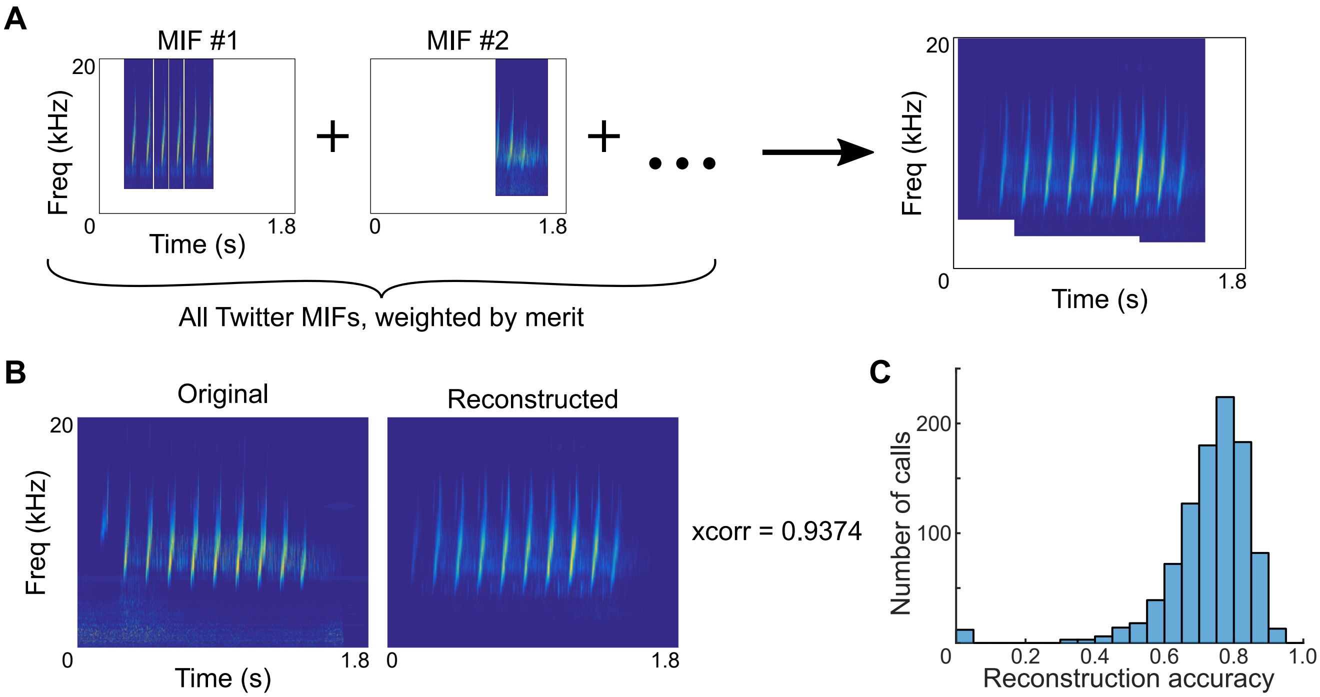

Supplementary Figure 5: Reconstruction of twitter calls using only twitter MIFs. (A) Cochleagrams of MIFs were placed at the time points at which MIFs were detected within a sample twitter call. All MIF cochleagrams were then summed, weighted by their log-likelihood ratios. **(B)** Cochleagrams of and example original twitter call and its reconstructed version. **(C)** Histogram of the reconstruction accuracy of 1000 twitter calls.

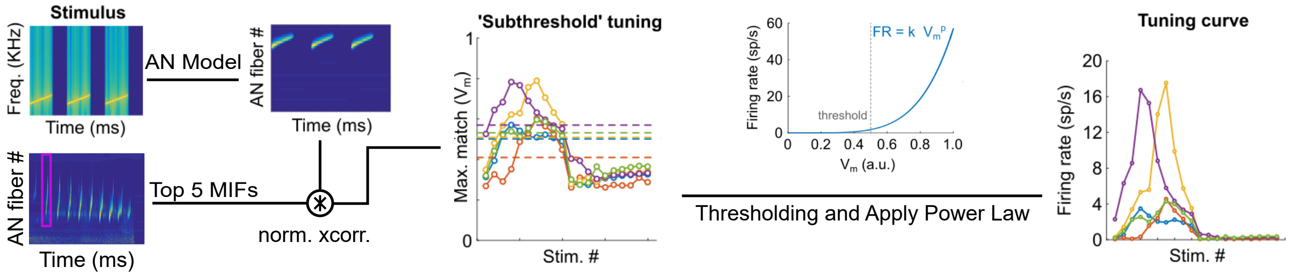

Supplementary Figure 6: Simulation of putative MIF-neuron tuning properties. The responses of MIFs to cochleagrams of commonly used auditory stimuli were taken to be the maximum value of the normalized cross-correlation function. A power law nonlinearity was applied to this value to obtain 'tuning curves' of the MIF-neurons to these stimuli.

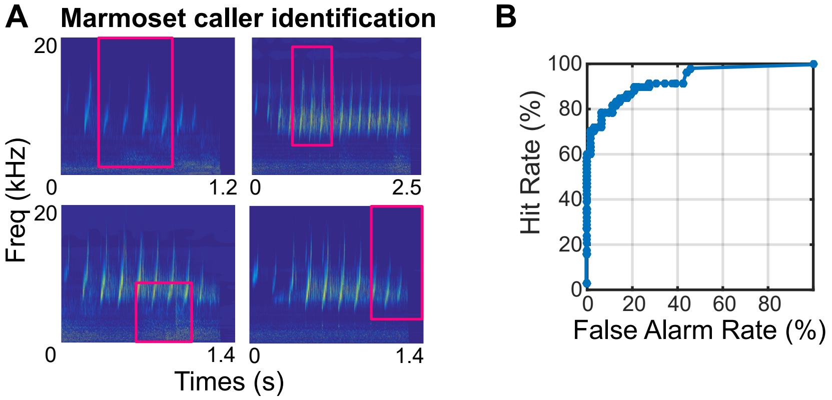

Supplementary Figure 7: Caller identification for a pair of marmoset callers with overlapping dominant frequencies. (A) MIFs for caller identification. **(B)** ROC curve for caller identification.
